# Short-term continuous light exposure induces hippocampal rhythmic and functional alterations: a multi-timepoint metabolomics study

**DOI:** 10.3389/fmolb.2026.1753977

**Published:** 2026-02-06

**Authors:** Xiuxuan Wu, Guanyu Zhang, Shuai Wu, Yuyu Hu, Yongqiang Zhang, Li Zhang, Xi Li, Jun Li, Danfeng Yang

**Affiliations:** 1 School of Public Health, The Key Laboratory of Environmental Pollution Monitoring and Disease Control, Ministry of Education, Guizhou Medical University, Guiyang, China; 2 Academy of Military Medical Sciences, Academy of Military Sciences, Tianjin, China

**Keywords:** circadian rhythm, cognitive dysfunction, continuous light exposure, hippocampus, metabolic rhythm disruption, metabolomics

## Abstract

**Introduction:**

Short-term continuous light exposure disrupts circadian rhythms and impairs cognitive function, yet its specific effects on dynamic metabolic oscillations within the hippocampus remain unclear.

**Methods:**

This study investigated the impacts of short-term (7-day) continuous light exposure (LL) on hippocampal circadian rhythms, metabolome, and cognition in mice. We established an LL model and utilized behavioral assays, histopathology, qPCR for clock genes, and targeted time-series metabolomics (at ZT0, 6, 12, 18).

**Results:**

Our results showed that LL significantly disrupted the circadian oscillations of core clock genes, induced memory decline, and caused hippocampal neuronal abnormalities and neuroinflammation. Metabolomic analysis uncovered extensive remodeling of circadian metabolite rhythms, with a 56.9% reduction in rhythmic metabolites. We identified eight core rhythm-disrupted metabolites, among which the key inhibitory neurotransmitter GABA exhibited a pronounced phase delay (∼6.4 h) and reduced amplitude. Pathway analysis highlighted vitamin B6 metabolism as a central disrupted pathway.

**Discussion:**

These results illustrate that short-term continuous light exposure induces multi-level perturbations in the hippocampal circadian-metabolic network, with GABA and vitamin B6 metabolism as potential critical nodes linking circadian disruption to cognitive impairment. This study provides novel metabolomic insights and a descriptive foundation for understanding the mechanisms underlying light pollution-associated cognitive deficits.

## 1 Introduction

Circadian rhythms constitute an intrinsic timekeeping system that allows organisms to adapt to the 24-h day–night cycle, coordinating physiological activities, behavioral patterns, and metabolic processes to sustain the orderly progression of biological functions (Allada and Bass, 2021). The suprachiasmatic nucleus (SCN) of the hypothalamus serves as the regulatory hub, autonomously generating rhythms (Hastings et al., 2019) and perceiving external light signals via intrinsically photosensitive retinal ganglion cells (ipRGCs) (Zhang et al., 2021). This synchronizes rhythmic activities across peripheral tissues—including the liver, kidneys, and hippocampus—forming an integrated central–peripheral regulatory network that maintains homeostasis via hormonal (Blancas-Velazquez et al., 2023; Ramirez-Plascencia et al., 2025), metabolic (Reinke and Asher, 2019), and immune pathways (Zeng et al., 2024). However, modern lifestyles characterized by artificial nighttime illumination and shift work have rendered continuous light exposure a widespread disruptor of circadian organization. Satellite data confirm that light pollution is intensifying globally (Kyba et al., 2017). Such exposure impairs SCN function, suppresses oscillations of core clock genes such as *Bmal1* and *Clock*, and can abrogate rhythmicity entirely (Bumgarner and Nelson, 2021). It also disrupts sleep–wake cycles by inhibiting melatonin synthesis (Schwartz et al., 2009). Critically, this circadian disruption may further trigger inflammatory responses within the central nervous system, particularly through the activation of microglia—the brain’s resident immune cells—thereby exacerbating tissue damage and cognitive dysfunction (Meng et al., 2023). These effects are time-dependent: short-term light exposure induces gene phase shifts, whereas long-term exposure causes irreversible dysregulation of circadian networks, contributing to chronic conditions including obesity, type 2 diabetes, and cognitive decline (Naveed et al., 2024; Schrader et al., 2024). The correlation between cognitive decline and hippocampal dysfunction suggests that continuous light may target the hippocampal circadian system to impair memory.

The hippocampus, a brain region central to learning and memory, relies on finely tuned neural circuit function to support processes such as memory encoding. For instance, NMDA receptors in CA1 help maintain firing rate homeostasis (Ruggiero et al., 2025), underscoring the region’s sensitivity to both internal rhythmic and external metabolic influences. Hippocampal synaptic plasticity exhibits clear circadian variation: long-term potentiation (LTP) varies in induction efficiency across the day (Nakatsuka and Natsume, 2014). At the molecular level, phosphorylation of BMAL1 regulates synaptic localization and plasticity (Barone et al., 2023), intact BMAL1 expression is required for memory retrieval (Hasegawa et al., 2019), and *Per1* oscillations correlate with daytime memory consolidation (Bellfy et al., 2023)—confirming that circadian disruption directly compromises hippocampal cognition. Metabolically, the hippocampus is highly vulnerable to fluctuations: neurons depend on glucose availability, and loss of GLUT3 or glycolytic enzymes causes memory deficits (Li et al., 2023). Furthermore, circadian control of mitochondrial oxidative phosphorylation matches energy supply to the demands of synaptic remodeling (Xavier et al., 2016), indicating that metabolic homeostasis is essential for hippocampal function.

Although previous work has linked hippocampal metabolic changes to cognitive impairment—for example, altered cholesterol sulfate levels in rats with radiation-induced memory deficits (Guan et al., 2024)—most studies have relied on static, single-timepoint metabolite measurements, failing to capture dynamic rhythmic profiles. Thus, the role of metabolic rhythm disruption in cognitive decline remains unclear. Time-series metabolomics addresses this gap by sampling across multiple circadian time points (e.g., ZT0, ZT6, ZT12, ZT18) to quantify oscillatory parameters such as phase and amplitude, thereby identifying specific patterns of rhythm disruption. A recent study that profiled eight mouse tissues over 24 h successfully mapped tissue-specific metabolic rhythms and revealed intertissue coordination (Dyar et al., 2018), offering a validated framework for investigating hippocampal metabolic rhythms under continuous light. Therefore, the present study uses a mouse model of short-term continuous light exposure to examine changes in hippocampal clock gene expression, circadian metabolite dynamics, and cognitive behavior. We aimed to systematically characterize the multi-level disruptions induced by continuous light exposure by integrating analyses of hippocampal clock gene expression, circadian metabolite dynamics via time-series metabolomics, and cognitive behavior. Our specific objectives were to identify the core metabolic oscillations disrupted by LL, pinpoint key metabolites and pathways whose rhythmicity is most vulnerable, and explore how these circadian-metabolic disturbances correlate with neuroinflammation and cognitive dysfunction. This integrated approach provides insights into the underlying metabolic mechanisms of light-induced cognitive dysfunction.

## 2 Materials and methods

### 2.1 Animal subjects and tissue collection

Eight-week-old male C57BL/6J mice (Vital River Laboratory Animal Technology Co., Ltd., Beijing, China) were randomly allocated to either a normal 12-h light–dark cycle (LD) group or a continuous light exposure (LL) group (500 lux). Following 7 days of intervention, behavioral assays were performed (n = 10 per group, total n = 20). For molecular and histological analyses, separate cohorts of mice (not subjected to behavioral tests) were sacrificed by cervical dislocation at designated Zeitgeber Times (ZT). The whole hippocampus was rapidly dissected from each hemisphere on an ice-cold plate. Tissue for histological analysis (ZT12 only; n = 3 per group, total n = 6) was immediately fixed in 4% paraformaldehyde. Tissue for all biochemical assays was flash-frozen in liquid nitrogen and stored at −80 °C. Frozen samples were allocated as follows: for qPCR and metabolomics, tissues from all four time points (ZT0, ZT6, ZT12, ZT18; n = 4 per group per time point, total n = 32 per assay) were used; for ELISA, tissue from ZT12 only was analyzed (n = 6 per group, total n = 12). All animal experimental procedures were approved by the Committee on the Ethics of Animal Experiments of the Academy of Military Medical Science (Approval Code: IACUC-AMMS-04-2024-025).

### 2.2 Behavioral testing

Behavioral assessments were conducted on day 8 post-intervention from ZT2–ZT6 (10:00–14:00) to minimize circadian influences.

Novel Object Recognition: Mice underwent three phases in an open-field chamber monitored with a video tracking system (SuperMaze): adaptation (5 min in an empty arena), familiarization (5 min with two identical objects A), and testing (5 min with one object A replaced by a novel object B 24 h later). Exploration was defined as sniffing within 3 cm of an object or physical contact; climbing on or sitting against an object was not counted. A minimum criterion of ≥20 s total exploration during the test session was applied for inclusion; all animals met this criterion and none were excluded. The recognition index (RI) was calculated as: RI = (Time exploring novel object B)/(Time exploring objects A+ B). Exploration frequency (contacts) and duration were automatically scored by the SuperMaze software and verified manually.

Passive Avoidance Test: A light–dark box apparatus equipped with an electrified grid (0.3 mA), a sound-attenuating enclosure, and video tracking (SuperPAS) was used. During the training phase, entry into the dark compartment triggered a mild foot shock (0.3 mA). During the test phase (24 h after training), latency to re-enter the dark compartment, number of entries (errors), and movement trajectories were recorded.

### 2.3 Histopathology and immunohistochemistry

Tissue processing: Following immediate fixation in 4% paraformaldehyde, the hippocampal tissues were dehydrated through a graded ethanol series, cleared in xylene, embedded in paraffin, and sectioned at a thickness of 4 μm for subsequent staining.

H&E Staining: Sections were deparaffinized, stained with hematoxylin (5 min), differentiated, counterstained with eosin (30 s–1 min), dehydrated, cleared, and mounted for whole-slide imaging.

Nissl Staining: After deparaffinization, the sections were stained with methylene blue (10 min), differentiated until the Nissl bodies were distinct, treated with ammonium molybdate (5 min), washed, dehydrated, cleared, and mounted for scanning, and the number of neurons in the CA3 region under ×20 magnification was quantified using the cell counting tool in ImageJ.

Immunohistochemistry: Antigen retrieval was performed via microwave treatment in EDTA buffer (pH 8.0). Endogenous peroxidase activity was blocked with 3% H_2_O_2_ (20 min), and nonspecific binding was blocked with 10% serum (30 min). The sections were incubated with anti–IBA-1 (1:200–1:1,000) at 4 °C overnight, followed by incubation with an HRP-conjugated secondary antibody (1 h at room temperature). The signal was developed with DAB, the nuclei were counterstained with hematoxylin, and the slides were dehydrated, cleared, and scanned. Quantitative analysis was performed using ImageJ. At 20× magnification, the CA1 and CA3 subregions of the hippocampus were selected as regions of interest (ROIs). For each ROI, the mean fluorescence Intensity (MFI) was measured and normalized to the average MFI of the LD control group within the corresponding subregion. IBA-1-positive cells within the ROIs were manually counted; only intact cells with a clearly defined DAPI-stained nucleus and specific IBA-1 immunoreactivity were included.

### 2.4 Quantification of hippocampal cytokine levels by ELISA

The hippocampal concentrations of interleukin-1β (IL-1β) and tumor necrosis factor-α (TNF-α) were quantified using specific commercial sandwich ELISA kits. Briefly, frozen hippocampal tissues were homogenized in ice-cold PBS. The total protein concentration of each homogenate was determined via a bicinchoninic acid (BCA) assay. For cytokine measurement, samples and standards were processed strictly in accordance with the respective kit protocols. Absorbance was measured, and cytokine concentrations were interpolated from standard curves. Final data are expressed as picograms of cytokine per milligram of total protein (pg/mg protein).

### 2.5 Analysis of circadian gene expression

Total RNA was extracted from hippocampal tissue using Trizol, and the concentration was determined. Reverse transcription was performed via a commercial kit. Quantitative PCR was conducted via SYBR Green dye, with *β-actin* as the endogenous control. Relative expression levels were calculated via the 2^(–ΔΔCT) method. The PCR primer sequences are shown in Supplementary Table S1.

### 2.6 Hippocampus-targeted metabolomics

Metabolite extraction: After the sample was slowly thawed at 4 °C, an appropriate amount of the sample was added to a precooled methanol/acetonitrile/water mixture (2:2:1, v/v). The mixture was vortexed and mixed, sonicated at low temperature for 30 min, allowed to stand at −20 °C for 10 min, and then centrifuged at 14,000 × g for 20 min at 4 °C, after which the supernatant was vacuum dried. For mass spectrometry analysis, 100 c of acetonitrile aqueous mixture (acetonitrile: water = 1:1, v/v) was added for reconstitution, the mixture was vortexed, the mixture was centrifuged at 14,000 × g for 15 min at 4 °C, and the supernatant was collected for analysis.

Standard preparation: A mixed standard stock solution was prepared using various standards, the standard solution was diluted in gradient order to obtain a series of calibration solutions, and then each gradient solution was processed according to the sample preparation method for machine testing.

LC conditions: The samples were separated via an Agilent 1,290 Infinity LC ultrahigh-performance liquid chromatography system (UHPLC) HILIC and C18 chromatography columns; the HILIC chromatography column temperature was 35 °C; the flow rate was 0.3 mL/min; the injection volume was 2 μL; the mobile phase composition was 90% water+2 mM ammonium formate+10% acetonitrile, B: acetonitrile+0.4% formic acid; the gradient elution procedure was as follows: 0–1.0 min, 85% B; 1.0–3.0 min, B linearly changed from 85% to 80%; 3.0–4.0 min, 80% B; 4.0–6.0 min, B linearly changed from 80% to 70%; and 6.0–10.0 min, B linearly changed from 70% to 50%; 10–15 min, B remained at 50%; 15.5–15.6 min, B linearly changed from 50% to 85%; and from 15.6 to 23 min, B remained at 85%. C18 chromatographic column temperature, 40 °C; flow rate, 0.4 mL/min; injection volume, 2 μL; mobile phase composition, A: water+5 mM ammonium acetate; B: 99.5% acetonitrile. The gradient elution procedure was as follows: 0–5 min, B linearly changed from 5% to 60%; 5–11 min, B linearly changed from 60% to 100%; 11–13 min, B remained at 100%; 13–13.1 min, B linearly changed from 100% to 5%; and 13.1–16 min, Maintained at 5%. The sample was placed in a 4 °C automatic sampler throughout the entire analysis process. To avoid the impact caused by fluctuations in instrument detection signals, a random sequence is used for continuous analysis of samples. The QC samples are inserted into the sample queue to monitor and evaluate the stability of the system and the reliability of the experimental data.

MS conditions: An AB 6500+QTRAP mass spectrometer (AB SCIEX) was used for mass spectrometry analysis. The ESI source conditions were as follows: source temperature: 580 °C, ion source gas 1 (GS1): 45, ion source gas 2 (GS2): 60, curtain gas (CUR): 35, and ion spray voltage (IS): +4500 V or −4500 V in positive or negative mode, respectively, and MRM mode monitoring was adopted.

### 2.7 Data analysis

All the statistical analyses were performed with SPSS 25.0 and R software (version 4.4.3), with the significance level set at α = 0.05 (*P* < 0.05 was considered statistically significant). Graphs were generated via specific tools on the basis of their types: bar graphs for behavioral indicators, Nissl body counts, quantitative immunofluorescence results (IBA-1 MFI and cell counts), and inflammatory cytokine (IL-1β and TNF-α) levels were plotted with GraphPad Prism 10.1.2, whereas other graphs were created by corresponding R packages or the MetaboAnalyst 6.0 platform.

#### 2.7.1 Data distribution test and intergroup difference analysis

The normality of all quantitative data was assessed using the Shapiro–Wilk test. Normally distributed data are presented as the mean ± standard deviation and were compared using independent samples t-tests. Non-normally distributed data are presented as the median with interquartile range (IQR) and were compared using the Mann–Whitney *U* test. This approach was applied to analyze intergroup differences in behavioral indicators (e.g., recognition index, exploration time and frequency, incubation period, number of errors) and pathological quantitative indicators (e.g., Nissl body count, IBA-1 mean fluorescence intensity and cell counts, cytokine levels). Effect sizes are reported alongside *p*-values, using *Cohen’s d* for t-tests and the rank-biserial correlation (*r*) for Mann–Whitney *U* tests.

#### 2.7.2 Metabolomic data processing and differential screening

Multivariate analysis: Principal component analysis (PCA), partial least squares-discriminant analysis (PLS-DA), and time-point-specific orthogonal partial least squares-discriminant analysis (OPLS-DA) were performed via the MetaboAnalyst 6.0 platform to evaluate the global metabolic pattern separation and time-specific metabolic differences between the LD and LL groups. A permutation test (permutation number = 1,000) was used to verify the reliability of the OPLS-DA model. Visualization graphs for PCA/PLS-DA/OPLS-DA were directly generated via MetaboAnalyst 6.0.

Differentially abundant metabolite screening: Global differentially abundant metabolites were screened with the criteria of “fold change *(FC) > 1.2* and *p < 0.05*”. Time-point-specific differentially abundant metabolites were screened with the criteria of “variable importance in projection *(VIP) > 1.5* and *p < 0.05*”.

#### 2.7.3 Circadian rhythm characteristic analysis

Rhythmicity screening: Circadian rhythmicity was first assessed for all targets (metabolites and clock genes) in the LD control group using the JTK_CYCLE algorithm. Only targets exhibiting significant circadian oscillations (*p < 0.05*) under LD conditions were retained for subsequent comparative rhythm analysis.

Rhythm parameter quantification: For the rhythmically significant targets, a mixed-effects model was fitted using the R package lme4. The model included interaction terms Group × cos (2πt/24) + Group × sin (2πt/24) (where t represents circadian time) to assess differences in rhythm parameters (phase, amplitude) between the LD and LL groups. A significant interaction term (*p < 0.05*) indicated a significant intergroup difference in circadian characteristics.

Visualization: Polar plots and amplitude heatmaps of core rhythmic metabolites were generated using ggplot2. Oscillation curves of rhythmic clock genes and metabolites were plotted using ggpubr.

#### 2.7.4 Pathway enrichment analysis

Pathway enrichment analysis of differentially abundant metabolites and core rhythm-disrupted metabolites was performed based on the KEGG database using the MetaboAnalyst 6.0 platform. In this study, an FDR-adjusted *p* value *(FDR) < 0.05* was set as the threshold for determining significantly enriched pathways. Bar graphs and bubble plots for pathway enrichment were automatically generated by the platform.

#### 2.7.5 Other analyses and visualization

Time series clustering heatmaps and intergroup expression trend graphs of metabolites were plotted via the R packages pheatmap and ggplot2.

Venn diagrams for the intersection of differentially abundant metabolites across multiple time points were drawn via the R package VennDiagram.

## 3 Results

### 3.1 Short-term continuous light exposure disrupts hippocampal circadian rhythms and leads to memory decline

**FIGURE 1:**
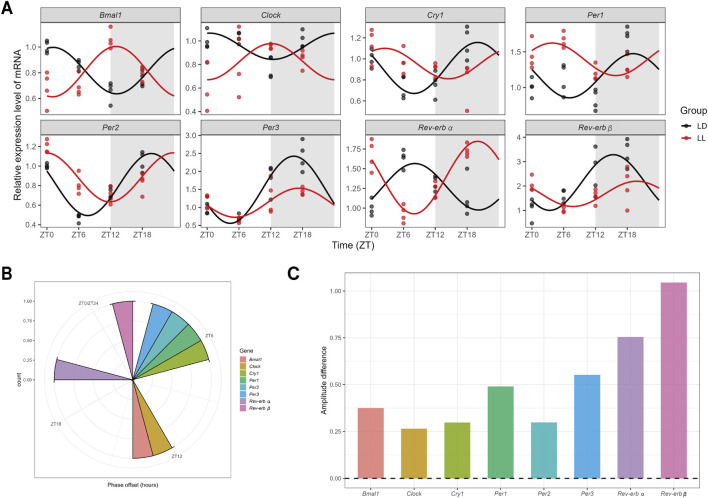
Short-term continuous light exposure disrupts hippocampal circadian rhythms in mice. **(A)** Effects of short-term continuous light exposure on diurnal oscillation patterns of gene expression in the mouse hippocampus. **(B)** Distribution of gene expression phase differences between the short-term continuous light exposure group and the control group. **(C)** Comparison of gene expression amplitude differences between the short-term continuous light exposure group and the control group. For all timepoint-specific analyses: n = 4 per group per time point (ZT0, ZT6, ZT12, ZT18).

To establish a model for studying the early neurobiological effects of continuous light, we first performed a pilot time-course experiment to define an exposure duration that induces robust hippocampal circadian disruption prior to long-term adaptation. Systematic comparison of 2-day (acute), 7-day (short-term), and 35-day (long-term) exposures revealed that 7-day continuous light elicited the most pronounced and widespread disruption of hippocampal core clock gene oscillations (Supplementary Table S2). We therefore defined 7 days as the “short-term” exposure window for subsequent investigation. Under this 7-day model, we next compared oscillation patterns between the LD and LL groups. For genes exhibiting significant 24-h rhythmicity in the LD control group, we fitted harmonic regression curves to compare their oscillation patterns between conditions, with visualization via the R package ggpubr. Results revealed that short-term continuous light significantly disrupted circadian oscillation patterns of core circadian genes in the hippocampus (Figure 1A), manifested as pronounced phase shifts (Figure 1B) and altered amplitudes (Figure 1C).

**FIGURE 2:**
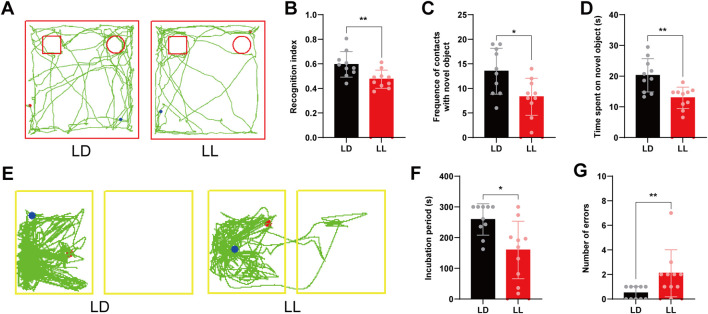
Short-term continuous light exposure induces memory impairment in mice (behavioral tests). **(A)** Representative movement trajectory from a mouse in the Novel Object Recognition test, selected based on a recognition index value closest to the group mean. **(B)** Recognition index. **(C)** Time exploring the novel object. **(D)** Number of contacts with the novel object. **(E)** Representative movement trajectory from a mouse in the Passive Avoidance test, selected based on an error count closest to the group median. **(F)** Latency to re-enter the dark compartment. **(G)** Number of errors. *p < 0.05, **p < 0.01. n = 10.

We next assessed the impact of this circadian disruption on memory. In the novel object recognition (NOR) test, total exploration time during training (LD: 28.38 ± 5.49 s; LL: 27.44 ± 4.29 s; *t (18) = 0.425, p = 0.676, Cohen’s d = 0.19*) and test phases (LD: 34.11 ± 7.77 s; LL: 27.40 ± 7.55 s; *t (18) = 1.958, p = 0.066, Cohen’s d = 0.88*) was comparable between groups (Supplementary Figure S1), indicating matched exploratory motivation. Under these conditions, LL mice exhibited significant memory impairment (exemplified by the movement trajectory in Figure 2A), as shown by a lower Recognition Index (LD: 0.596 ± 0.104; LL: 0.475 ± 0.074; *t (18) = 2.994, p = 0.008, Cohen’s d = 1.34*; Figure 2B), reduced time exploring the novel object (LD: 20.26 ± 5.42 s; LL: 12.95 ± 3.46 s; *t (18) = 3.599, p = 0.002, Cohen’s d = 1.61*; Figure 2C), and fewer contacts with it (LD: 13.50 ± 4.67; LL: 8.30 ± 3.77; *t (18) = 2.738, p = 0.014, Cohen’s d = 1.22*; Figure 2D). In the passive avoidance test, LL mice also performed worse (with a representative trajectory shown in Figure 2E), showing a significantly shorter latency to re-enter the dark chamber (*Median [IQR]*: LL = 180.32 [148.89] s, LD = 280.57 [75.79] s; Mann-Whitney *U = 17.5, p = 0.014, r = -0.55*; Figure 2F) and more errors during the test (Median [IQR]: LL = 2.0 [1.25], LD = 0.5 [1.0]; Mann-Whitney *U = 15.0, p = 0.005, r = -0.62*; Figure 2G).

### 3.2 Continuous light exposure induces hippocampal neuronal alterations and concomitant neuroinflammation

**FIGURE 3:**
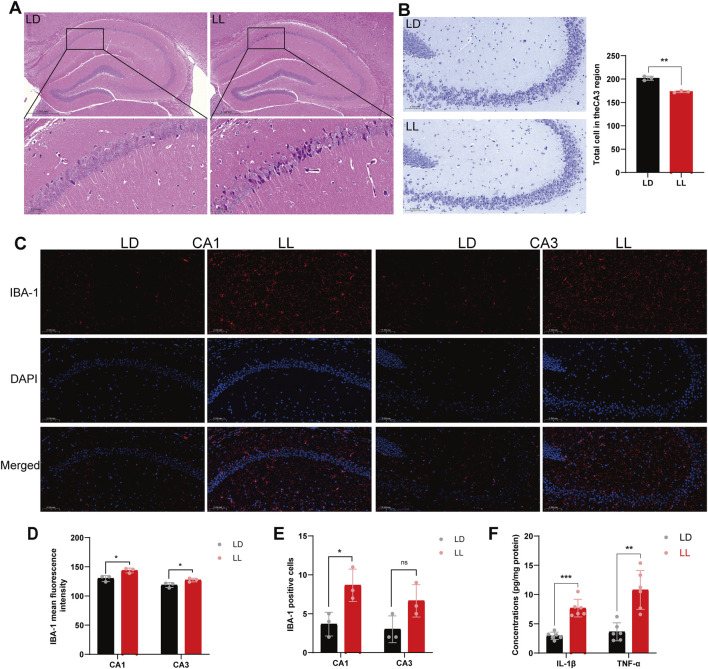
Short-term continuous light exposure induces hippocampal structural damage and neuroinflammation in mice. **(A)** H&E staining of mouse hippocampal tissue; ×6 magnification: scale bar = 0.5 mm; ×40 magnification: scale bar = 0.05 mm. **(B)** Nissl staining of the CA3 region in mouse hippocampal tissue (20×); scale bar = 0.1 mm **p < 0.01, n = 3. **(C)** Immunofluorescence staining for IBA-1 in mouse hippocampal tissue (20×); scale bar = 0.05 mm. The red fluorescence represents IBA-1, and blue fluorescence represents DAPI. **(D)** Quantitative analysis of IBA-1 mean fluorescence intensity (MFI) in the hippocampal CA1 and CA3 subregions. *P < 0.05, n = 3. **(E)** Quantification of IBA-1-positive cell numbers in the hippocampal CA1 and CA3 subregions. *p < 0.05, ns p > 0.05, n = 3. **(F)** Concentrations of the pro-inflammatory cytokines IL-1β and TNF-α in hippocampal tissue. **p < 0.01, ***p < 0.001, n = 6.

To investigate the structural and pathological underpinnings of the observed memory impairment, we examined neuronal integrity and neuroinflammatory status in the hippocampus. Histological examination revealed signs of neuronal alterations in LL mice, including shrinkage and nuclear condensation in the CA1 region (Figure 3A). Quantification showed a significant reduction in Nissl bodies—indicating diminished neuronal metabolic activity or number—in the CA3 region (LD: 201.67 ± 4.51; LL: 173.00 ± 2.00; *t (4) = 10.066, p = 0.001, Cohen’s d = 8.22*; Figure 3B). In parallel, a pronounced neuroinflammatory response was evident. Immunofluorescence revealed significantly elevated IBA-1 immunoreactivity (a marker of microglial activation) in the hippocampus of LL mice (Figure 3C). Quantitative analysis confirmed higher mean fluorescence intensity in both CA1 (LD: 130.06 ± 5.10; LL: 143.79 ± 4.15; *t (4) = -3.617, p = 0.022, Cohen’s d = -2.95*) and CA3 (LD: 118.80 ± 4.25; LL: 127.41 ± 3.11; *t (4) = -2.835, p = 0.047, Cohen’s d = -2.32*) (Figure 3D), coupled with an increased number of IBA-1–positive cells in CA1 (LD: 3.67 ± 1.53; LL: 8.67 ± 2.08; *t (4) = -3.354, p = 0.028, Cohen’s d = -2.74*). A similar increasing trend in CA3 did not reach statistical significance (Figure 3E). Biochemical analysis further confirmed a pronounced pro-inflammatory state, with hippocampal levels of IL-1β (LD: 2.95 ± 0.62 pg/mg protein; LL: 7.68 ± 1.51 pg/mg protein; *t (10) = -7.102, p < 0.001, Cohen’s d = -4.10*) and TNF-α (LD: 3.63 ± 1.54 pg/mg protein; LL: 10.78 ± 3.31 pg/mg protein; *t (10) = -4.800, p = 0.001, Cohen’s d = -2.77*) significantly elevated in the LL group compared to controls (Figure 3F).

### 3.3 Continuous light exposure induces global metabolic disruption in the hippocampus

**FIGURE 4:**
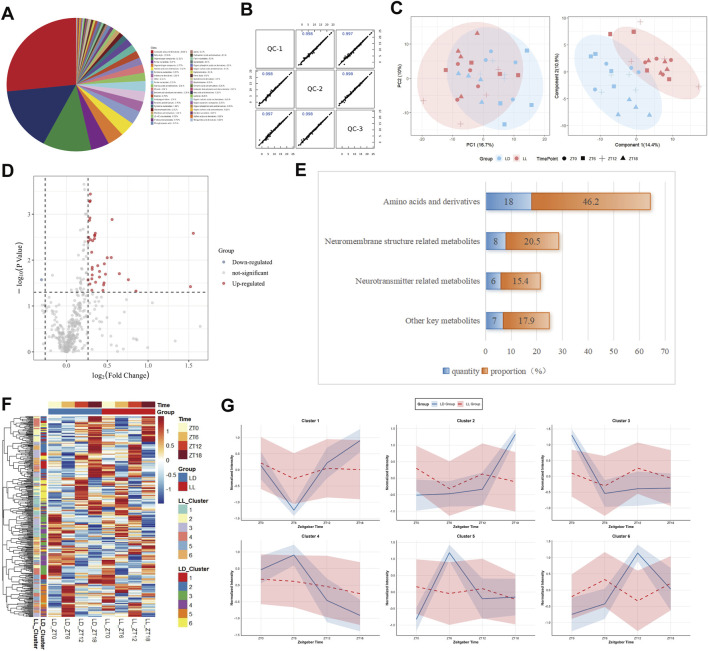
Effects of circadian rhythm disorders on spatiotemporal patterns of the mouse hippocampal metabolome. **(A)** The proportion of identified metabolites in each chemical classification. **(B)** QC sample correlation chart. **(C)** Principal component analysis showing significant separation of hippocampal metabolic patterns between the LD and LL groups and a PLS-DA plot of LD and LL time series metabolomics. **(D)** Volcano plot of differentially abundant metabolites in the hippocampus between the LD and LL groups at the four merged time points (*FC > 1.2, p < 0.05*). **(E)** Stacked bar chart of differentially abundant metabolites in the hippocampus between the LD and LL groups by functional category. **(F)** Metabolite time series clustering heatmap. **(G)** Time expression trend analysis of metabolites in each group. For metabolomics analyses: n = 4 per group per time point (ZT0, ZT6, ZT12, ZT18).

To investigate the metabolic basis of light-induced hippocampal dysfunction, we performed targeted metabolomics at four circadian time points (ZT0, ZT6, ZT12, and ZT18). We identified 398 metabolites, predominantly carboxylic acids and derivatives (26.63%) and fatty acyls (15.58%) (Figure 4A; Supplementary Table S3). QC samples were highly correlated (*r > 0.99*), confirming data reliability (Figure 4B; Supplementary Figures S2–S6). PCA and PLS-DA revealed distinct separation between the LD and LL groups across all time points (Figure 4C), indicating systemic metabolic disruption. We identified 39 differentially abundant metabolites (*FC > 1.2, p < 0.05*) between groups (Figure 4D; Supplementary Table S4), primarily comprising amino acids and derivatives, neuronal membrane components, and neurotransmitter-related metabolites (Figure 4E). Time-series clustering revealed markedly different oscillation patterns for 398 metabolites between groups (Figure 4F), with cluster trend plots showing category-specific expression differences (Figure 4G).

### 3.4 Identification of rhythm-disrupted metabolites and GABA dysregulation

**FIGURE 5:**
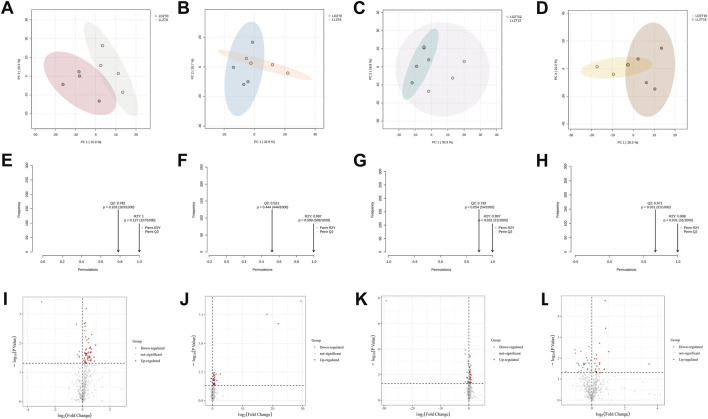
Differentially abundant metabolite screening and group discrimination analysis of the hippocampus at different time points. **(A–D)** PCA score plots: Hippocampal metabolites between the LD and LL groups (**(A)**: ZT0, **(B)** ZT6, **(C)** ZT12, **(D)** ZT18); **(E–H)** OPLS-DA permutation plots: Same as above (**(E)**: ZT0, **(F)** ZT6, **(G)** ZT12, **(H)** ZT18); **(I–L)** Volcano plots: Differential hippocampal metabolites between the LD and LL groups (**(I)**: ZT0, **(J)** ZT6, **(K)** ZT12, **(L)**: ZT18). For all time-point-specific analyses: n = 4 per group per time point.

To characterize time-specific metabolic perturbations, we conducted differential analysis at individual time points. PCA revealed metabolic pattern separation between the LD and LL groups at ZT0, ZT6, and ZT18, with increased dispersion in LL samples at ZT12 (Figures 5A–D). OPLS-DA models confirmed excellent separation at all time points (Figures 5E–H). We identified 160 timepoint-specific differentially abundant metabolites using the criteria of *VIP > 1.5* and *p < 0.05* (Figures 5I–L; Supplementary Tables S5-S8).

**FIGURE 6:**
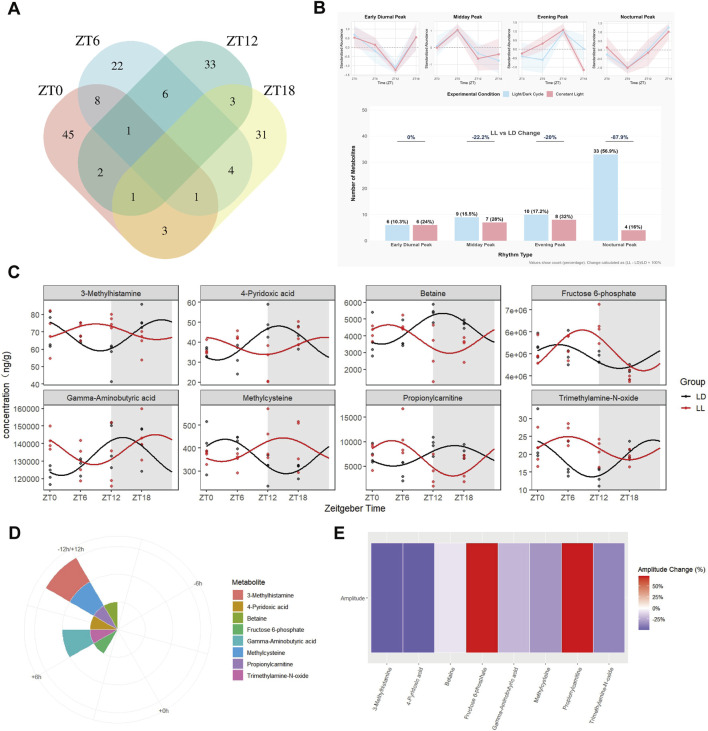
Cross-time point associations of differential hippocampal metabolites and characteristic analysis of core rhythmic differential molecules. **(A)** Venn diagram of intersections of differentially abundant metabolites at four time points (ZT0--ZT18); **(B)** Analysis of rhythmic metabolite types in the LD and LL groups; **(C)** Comparison plot of oscillation patterns of 8 core rhythmic differentially abundant metabolites in the LD and LL groups (fitted by a mixed-effects model); **(D)** Polar plot of phase differences (LL group phase–LD group phase) of 8 core rhythmic differentially abundant metabolites; **(E)** Heatmap of amplitude change percentage of 8 core rhythmic differentially abundant metabolites (amplitude change percentage = (LL group amplitude–LD group amplitude)/LD group amplitude×100%). Metabolomic data were derived from n = 4 per group per time point.

Venn analysis revealed no common differentially abundant metabolites across all four time points, with three metabolites shared among three time points (Figure 6A). Continuous light exposure reduced the number of rhythmic metabolites from 58 to 25 (56.9% decrease) and disrupted the nocturnal peak-dominated distribution pattern (Figure 6B; Supplementary Tables S9, S10). Using LD rhythmic metabolites as a reference, a mixed-effects model identified eight core circadian-disrupted metabolites: 3-Methylhistamine, 4-Pyridoxic acid, Betaine, Fructose 6-phosphate, Gamma-Aminobutyric acid (GABA), Methylcysteine, Propionylcarnitine, and Trimethylamine-N-oxide. The inhibitory neurotransmitter GABA exhibited a 6.4-h phase delay and 21% amplitude reduction in LL mice (Figure 6C; Supplementary Table S11). Polar plots and amplitude heatmaps confirmed phase shifts and amplitude alterations in other core metabolites (Figures 6D,E).

### 3.5 Vitamin B6 metabolism emerges as a key disrupted pathway

**FIGURE 7:**
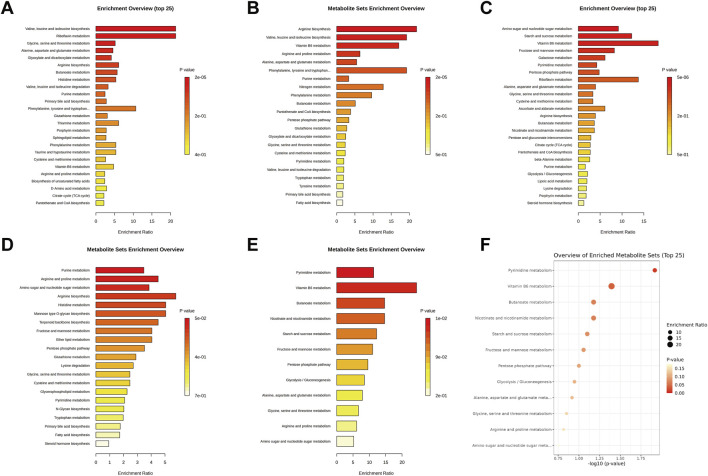
KEGG pathway enrichment analysis of hippocampal differentially abundant metabolites and rhythmic-disordered metabolites under constant light exposure. **(A)** Pathway enrichment of differentially abundant metabolites at ZT0; **(B)** Pathway enrichment of differentially abundant metabolites at ZT6; **(C)** Pathway enrichment of differentially abundant metabolites at ZT12; **(D)** Pathway enrichment of differentially abundant metabolites at ZT18; **(E)** Bar plot of pathway enrichment for 8 rhythm-disrupted metabolites; **(F)** Bubble plot of pathway enrichment for 8 rhythm-disrupted metabolites. All P-values shown in this figure have been adjusted for multiple comparisons using the False Discovery Rate (*FDR*) method.

KEGG enrichment analysis of timepoint-specific differentially abundant metabolites revealed distinct pathway perturbations across the circadian cycle: valine, leucine, and isoleucine biosynthesis was significantly enriched at ZT0 (*FDR = 0.0013*, Figure 7A; Supplementary Table S12), arginine biosynthesis at ZT6 (*FDR = 0.0015*, Figure 7B; Supplementary Table S13), and amino sugar and nucleotide sugar metabolism (*FDR = 0.0004*), starch and sucrose metabolism (*FDR = 0.0090*), and vitamin B6 metabolism (*FDR = 0.0112*) at ZT12 (Figure 7C; Supplementary Table S14). No pathway met the significance threshold at ZT18 (Figure 7D; Supplementary Table S15). Notably, when focusing on the core set of 8 metabolites with disrupted circadian rhythms, vitamin B6 metabolism again emerged as the top associated pathway (*raw p = 0.023*), consistent with its significant enrichment at ZT12—though this association did not survive strict FDR correction for multiple testing (*FDR = 0.804*, Figures 7E,F; Supplementary Table S16). This convergent evidence points to vitamin B6 metabolism as a pathway particularly vulnerable to continuous light exposure.

## 4 Discussion

Light pollution has emerged as an increasingly prevalent environmental stressor in modern society, and its disruption of the circadian system is linked to an increased risk of various neurological disorders (Voigt et al., 2024). Previous studies monitoring overall activity/rest rhythms in animals have demonstrated significant impacts of light disturbance on behavioral circadian patterns (Walker et al., 2020). In this context, the present study delves further into specific tissues, aiming to systematically investigate how oscillations of the internal “molecular clock” and rhythms of the “metabolic clock” in the hippocampus—a region closely linked to cognition—respond to early-life light exposure. To address this question, we first focused on establishing a short-term exposure model capable of capturing initial pathogenic effects. By systematically comparing continuous light exposure for 2 days (acute), 7 days (short-term), and 35 days (long-term), we found that 7-day (short-term) exposure induced the most pronounced and widespread disruption in the oscillation of core clock genes in the hippocampus, while avoiding complex adaptive changes that might arise from longer-term exposure. Therefore, this study defined “7 days” as the “short-term” exposure window and integrated behavioral analysis, multi-timepoint metabolomics, and molecular biology assays to systematically characterize multi-level disturbances in the hippocampus under short-term continuous light exposure.

Based on the aforementioned model, we found that short-term continuous light exposure first significantly disrupted oscillation patterns of core clock genes in the hippocampus, inducing notable phase shifts and amplitude alterations (Figure 1). Stable rhythmic expression of hippocampal core clock genes serves as a crucial molecular foundation for maintaining synaptic plasticity and memory function (Hasegawa et al., 2019; Barone et al., 2023; Bellfy et al., 2023), which relies on coordinated interactions between the *Clock*/*Bmal1* positive feedback loop and the *Per*/*Cry* and *Rev-erb α*/*β* negative feedback loops (Bugge et al., 2012). This study discovered that even short-term continuous light exposure significantly disturbed rhythmic expression of hippocampal clock genes, leading to memory impairment in mice. This observation aligns with findings from other circadian disruption models, such as sleep deprivation (Jiao et al., 2022), which also demonstrated cognitive deficits. The functional specificity of the hippocampus as a cognition-related brain region may stem from its dual regulatory requirement—maintaining intrinsic rhythms while responding to central zeitgeber signals (Liu et al., 2024)—potentially rendering it more vulnerable to external light disturbances. Importantly, the vulnerability of hippocampal rhythmic function to short-term continuous light exposure resonates with the finding that “circadian disruption is preferentially associated with reduced hippocampal volume and mediates the risk of cognitive decline” (Luo et al., 2025), further suggesting the hippocampus is a potential early target through which rhythm disruption triggers cognitive impairment.

To investigate the biochemical basis of early functional impairment, we further employed time-series metabolomic analysis. Disruption of core clock genes has been shown to directly regulate circadian oscillations of metabolic genes, thereby driving rhythmic expression of metabolites (Schrader et al., 2024). Our time-series metabolomic analysis confirmed that this top-down regulatory mechanism was systematically disrupted under light interference (Figure 4). PCA revealed clear separation between control and light-exposed groups, indicating an imbalance in overall metabolic homeostasis. Subsequent screening identified 39 differential metabolites, primarily involving amino acids and their derivatives, neuron membrane-related lipids, and neurotransmitter-related metabolites. This suggests selective disturbance of specific metabolic pathways rather than a generalized effect. Among these, elevated levels of metabolites related to neuronal membrane structure hinted at disrupted membrane homeostasis (Yue et al., 2020), while a significant decrease in suberylcarnitine—a key intermediate in mitochondrial β-oxidation—reflected impaired energy metabolism (Guerreiro et al., 2018). Continuous light exposure reduced rhythmic metabolites by 56.9%, and the proportion of metabolites peaking at night dropped from 56.9% to 16%. Given that these metabolites are primarily involved in memory-related processes such as cellular repair and energy storage (Hepler et al., 2022), their sharp decline may directly undermine the metabolic foundation required for cognitive function. Our analysis further pinpointed a highly specific axis of perturbation. From the multitude of rhythmically abnormal metabolites, we identified eight core metabolites with disrupted rhythms. Notably, the rhythmic disturbance of GABA—the major inhibitory neurotransmitter in the hippocampus—was particularly critical (Figure 6C). This is not only because GABA is a core neurotransmitter for maintaining hippocampal excitation/inhibition balance and memory consolidation (Gao et al., 2014; Sohal and Rubenstein, 2019; Wu et al., 2024), but also because its endogenous peak in the control group was precisely timed to the critical period for memory consolidation (ZT12-18) (Maddaloni et al., 2024). In contrast, light treatment caused a significant phase delay (approximately 6.4 h) and a reduction in amplitude (21%). This dysregulation of a core neurotransmitter rhythm within a critical time window likely directly disrupts the precise neurochemical timing and circuit stability necessary for memory consolidation. It is important to emphasize that the current measurements, based on bulk hippocampal tissue, cannot distinguish whether the observed alterations in GABA rhythm stem from an imbalance between synthesis and degradation, specific contributions of neurons versus glial cells, or heterogeneous distribution across hippocampal subregions. Precise characterization of these aspects awaits future investigations utilizing high-resolution techniques such as single-cell sequencing, spatial metabolomics, or *in vivo* microdialysis.

Importantly, this study identified vitamin B6 metabolism as a significantly disrupted pathway, suggesting it may serve as an upstream regulatory node for GABA phase abnormality. This provides a crucial clue for interpreting how continuous light exposure (LL) disrupts the rhythmicity of hippocampal γ-aminobutyric acid (GABA)ergic transmission. This potential regulatory pathway originates from the global control of metabolic processes by the circadian clock: existing research confirms that core clock proteins (e.g., CLOCK/BMAL1) can rhythmically bind to promoter regions of a vast number of target genes across the genome, with genes related to metabolic pathways being among their core regulatory targets (Koike et al., 2012). This implies that LL-induced circadian disruption in the hippocampus may broadly impair the rhythmic expression of downstream metabolic enzymes, thereby affecting the metabolic homeostasis of vitamin B6. The active form of vitamin B6—pyridoxal-5′-phosphate (PLP)—is an essential cofactor for glutamate decarboxylase (GAD), the rate-limiting enzyme in GABA synthesis; classic biochemical studies clearly establish that the covalent binding of PLP to the active site of GAD is a prerequisite for initiating the enzymatic reaction of GABA synthesis (Ueland et al., 2015). Although the circadian rhythm of GAD activity in the hippocampus has not been directly demonstrated, in the central circadian clock (the suprachiasmatic nucleus, SCN), the synthesis, release, and receptor function of GABA signals are tightly and dynamically coupled with the core clock, providing a key theoretical paradigm for understanding the clock-regulated mode of neurotransmitter synthesis in other brain regions (Albers et al., 2017). Therefore, the LL-induced disruption of vitamin B6 metabolism likely interferes with the rhythmic foundation of GABA synthesis by disturbing the homeostatic supply of PLP. This “metabolism-neurotransmitter” coupling perspective not only precisely links the external stimulus of environmental light with neurochemical changes within the hippocampus but also provides critical molecular evidence for deciphering how light exposure influences neurotransmitter rhythms through time-specific metabolic regulation. It should be emphasized that the proposed regulatory model remains a testable hypothesis, grounded in our correlative findings and established biochemical principles; definitive establishment of causality will require future validation through targeted interventional studies.

The disruption in the vitamin B6 pathway and GABA rhythmicity provides a critical entry point for understanding the complex pathological phenotypes observed in the hippocampus under light exposure. First, at the behavioral level, the phase delay in GABA rhythm is congruent with the observed memory impairment, offering a direct and plausible neurophysiological basis for explaining the impaired recent memory exhibited by mice under continuous light exposure in the Novel Object Recognition and Passive Avoidance tests. We selected these two tests precisely because they effectively capture hippocampal-dependent memory abnormalities sensitive to temporal cues: performance in the Novel Object Recognition test highly depends on the time delay between learning and testing (Clark et al., 2000), while the Passive Avoidance test can accurately reflect memory consolidation within a specific time window (Taubenfeld et al., 2001). Temporal analysis further revealed circadian phase-specific metabolic disturbances: the most significant differences occurred at ZT0—a key time node in entrainment mechanisms (Van Reeth and Turek, 1992)—and at ZT12—a critical window for memory consolidation (Maddaloni et al., 2024). These disturbances correspond to disruptions in time-keeping mechanisms and abnormal energy supply, respectively, thereby explaining from a metabolic perspective “why light exposure preferentially impairs specific cognitive functions.” Second, at the cellular microenvironment level, this concurrent disruption of rhythms and metabolism is closely associated with an imbalance in neuroimmune homeostasis. One important function of the circadian system is to suppress unnecessary inflammatory responses during the rest phase by regulating pathways such as NF-κB (Spengler et al., 2012). Consistent with this theoretical expectation, we synchronously observed transformation of microglia into an activated state and a significant increase in levels of pro-inflammatory cytokines IL-1β and TNF-α in hippocampal tissues with disrupted circadian rhythms—further supporting the notion that circadian disruption may lead to breakdown of neuroimmune homeostasis and uncontrolled inflammatory responses. Notably, microglia, as the brain’s intrinsic immune sentinels, have their physiological functions and immune responses precisely regulated by the endogenous circadian clock (Guzmán-Ruiz et al., 2023). This makes them particularly sensitive to rhythm disturbances caused by light exposure, positioning them as key cells mediating early neuroinflammation. Furthermore, key products of microglial activation—IL-1β and TNF-α—have been confirmed to directly impair hippocampal synaptic plasticity and function (Pribiag and Stellwagen, 2013; Hoshino et al., 2017). This suggests that the inflammatory state we observed likely directly contributes to functional impairment of hippocampal circuits through these mechanisms. Subsequently, at the tissue structure level, the aforementioned functional disturbances are directly reflected in abnormal neuronal morphology. In the light-exposed group, neuronal shrinkage in the hippocampal CA1 region and reduced Nissl bodies in the CA3 region align with pathological changes induced by inflammatory stress. This process may be related to the “phagoptosis” mechanism mediated by activated microglia (Brown and Neher, 2014), wherein microglia can directly phagocytose viable neurons under inflammatory stress, leading to structural damage.

Our findings are suggestive of the possibility that continuous light exposure may trigger a hippocampal damage cascade: circadian rhythm disruption of clock genes may induce systemic metabolic rhythm alterations; abnormal GABA rhythms and vitamin B6 metabolic disturbances may represent key intermediate links, potentially affecting neurotransmitter homeostasis; and these changes may be associated with hippocampal structural abnormalities and cognitive decline. Additional metabolite alterations provide further clues: disrupted fructose-6-phosphate rhythms and enriched starch-sucrose metabolism pathways during ZT12 suggest potential temporal disruption of energy supply during memory consolidation windows (Shao et al., 2018; Landry et al., 2025); abnormal betaine rhythms may influence epigenetic modification processes (Li et al., 2015); and TMAO dysrhythmia coupled with altered butyrate metabolism suggests possible gut–brain axis involvement in neuroinflammation regulation (Matenchuk et al., 2020; Hoyles et al., 2021; Connell et al., 2022). Collectively, these findings support the concept that light exposure affects the hippocampus through multi-pathway, multi-level systemic perturbations, where temporally and functionally interconnected abnormalities form a potential network of action.

Furthermore, this study has several limitations. First and foremost, our findings establish correlative links between circadian-metabolic disruption and functional impairment; however, causal relationships remain to be definitively established. Future interventional studies (e.g., modulating vitamin B6 metabolism or GABAergic signaling) are needed to test the proposed pathways. Second, behavioral testing was conducted at a fixed circadian phase (ZT2–ZT6) to control for time-of-day variation. While this design robustly confirms a basal memory decline, it cannot reveal whether the endogenous circadian rhythm of memory performance itself is altered. Future studies employing multi-timepoint testing across the 24-h cycle are needed to address this question. Third, although we employed a short-term exposure model to capture initial disruptions, the observed effects could, in part, involve non-specific stress responses. Future work incorporating direct stress markers (e.g., corticosterone) would help distinguish circadian-specific effects from general stress. Finally, this study was conducted in male mice. Generalizability to females, whose circadian and metabolic systems may respond differently, requires explicit investigation in future studies.

## 5 Conclusion

In summary, this study demonstrates that short-term continuous light exposure is associated with multi-level disruptions in the mouse hippocampus. These disruptions include desynchronization of core circadian clock genes, systemic loss and phase alteration of metabolic rhythms, onset of neuroinflammation, neuronal structural abnormalities, and impairments in spatial and aversive memory. Notably, among the observed metabolic disturbances, the rhythm of the neurotransmitter GABA was significantly phase-delayed and dampened, and the vitamin B6 metabolism pathway was prominently dysregulated. Concurrent alterations were also noted in metabolites related to energy metabolism, methylation, and the gut-brain axis. Collectively, these results delineate a correlative framework in which environmental circadian disruption, metabolic rhythm disturbances, neuroinflammation, and cognitive decline co-occur in the hippocampus following short-term continuous light exposure. Future studies employing interventional designs are needed to establish causal relationships among these pathways. This work provides novel metabolomic clues and a descriptive foundation for further investigating the health risks associated with light pollution.

## Data Availability

The original contributions presented in the study are included in the article/Supplementary Material, further inquiries can be directed to the corresponding authors.
