# Supplementary material for: Short-term continuous light exposure induces hippocampal rhythmic and functional alterations: a multi-timepoint metabolomics study
Source: Front Mol Biosci. 2026 Feb 6;13:1753977. doi: 10.3389/fmolb.2026.1753977 (PMC12921413; doi:10.3389/fmolb.2026.1753977)
Supplement: Supplementary file 2 [file Supplementaryfile2.docx]

Supplementary Material


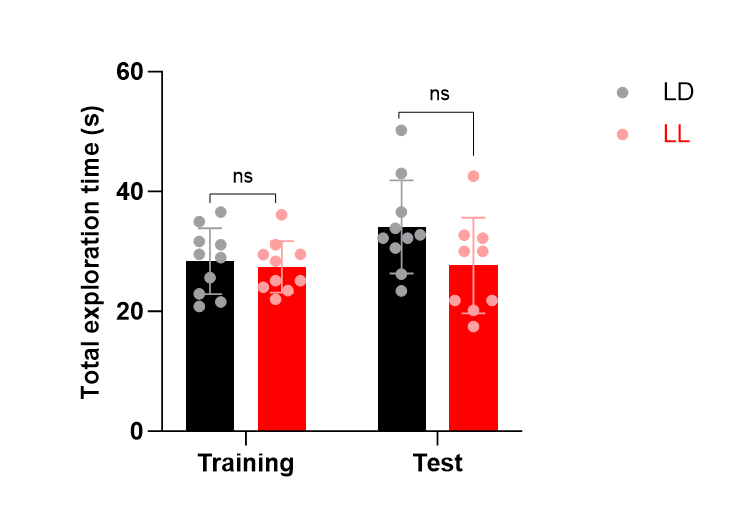


**Supplementary Figure 1.** Comparison of total exploration time during training and test phases of the novel object recognition test between LD and LL groups


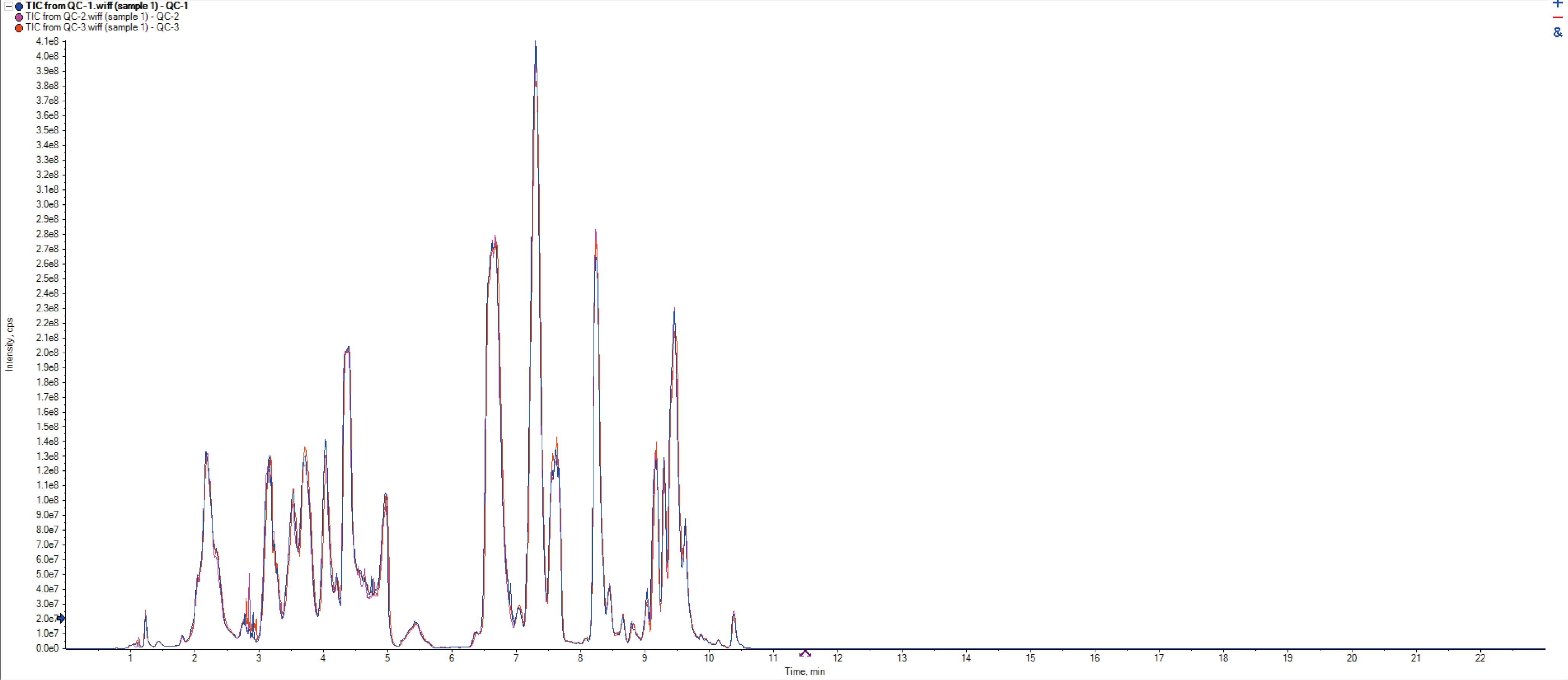


**Supplementary Figure 2.** Overlapping ion chromatogram of total ion current of Amide chromatographic column QC sample

**
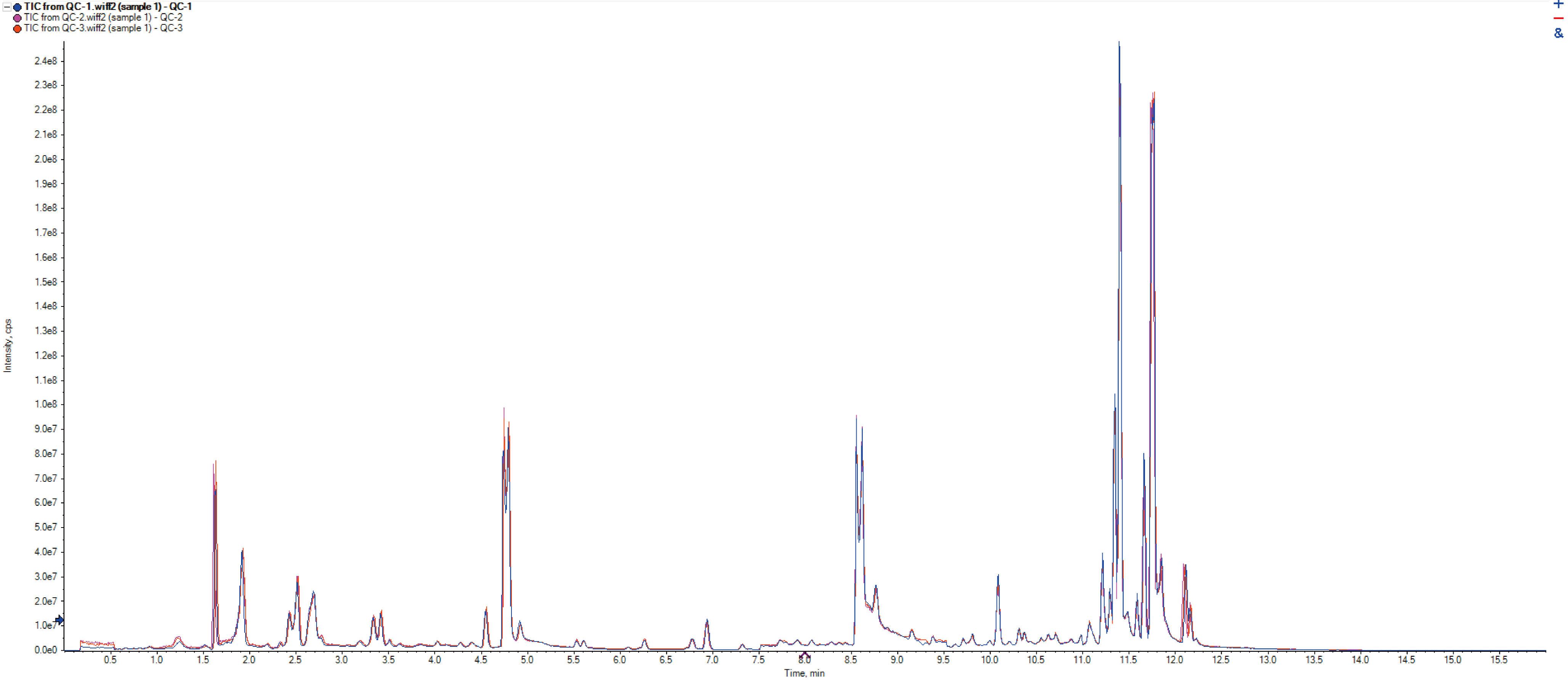
**

**Supplementary Figure 3.** Overlapping spectrum of total ion chromatogram of C18 chromatographic column QC sample


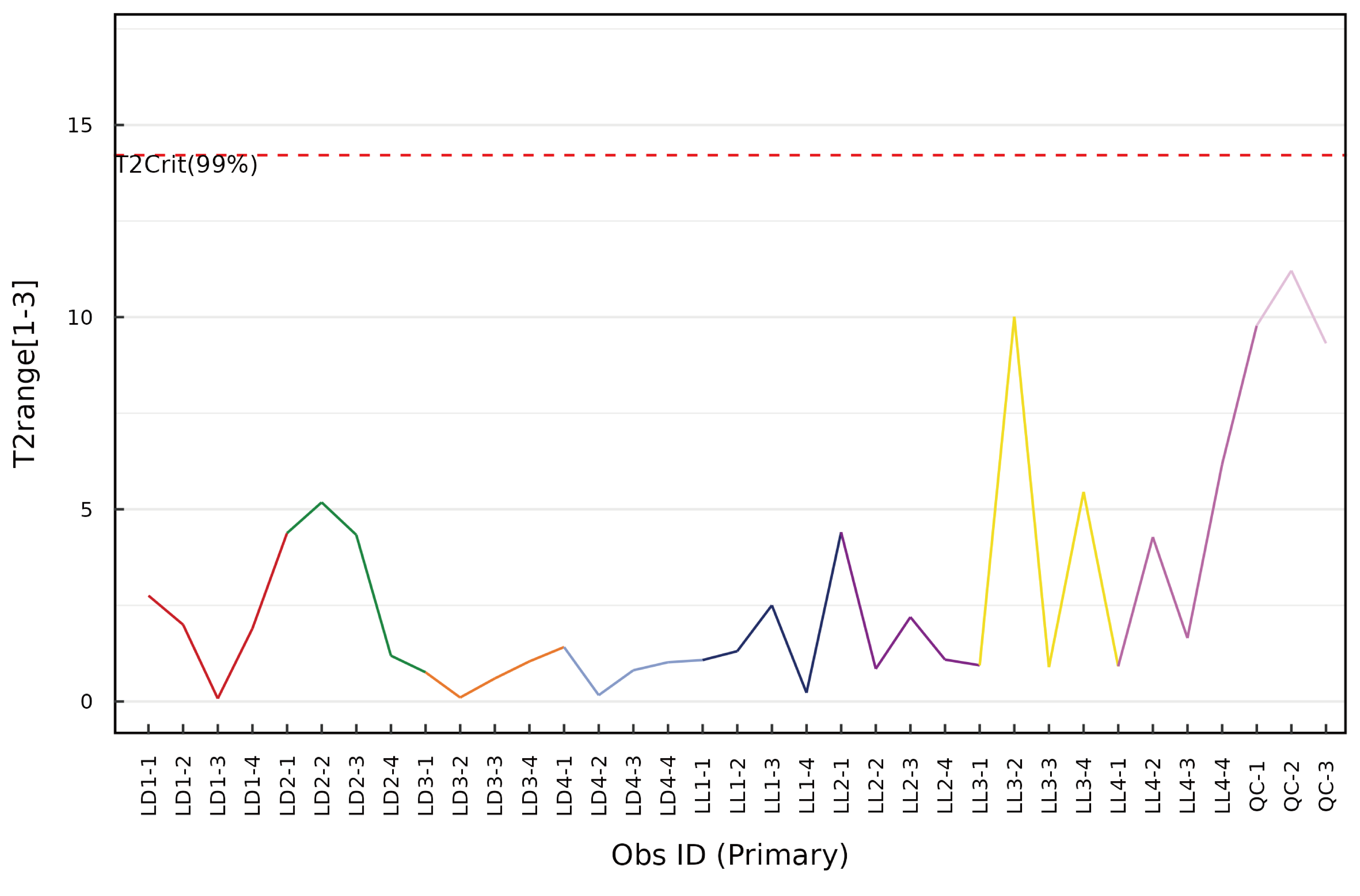


**Supplementary Figure 4.** Hotellings T2 plot of the overall sample


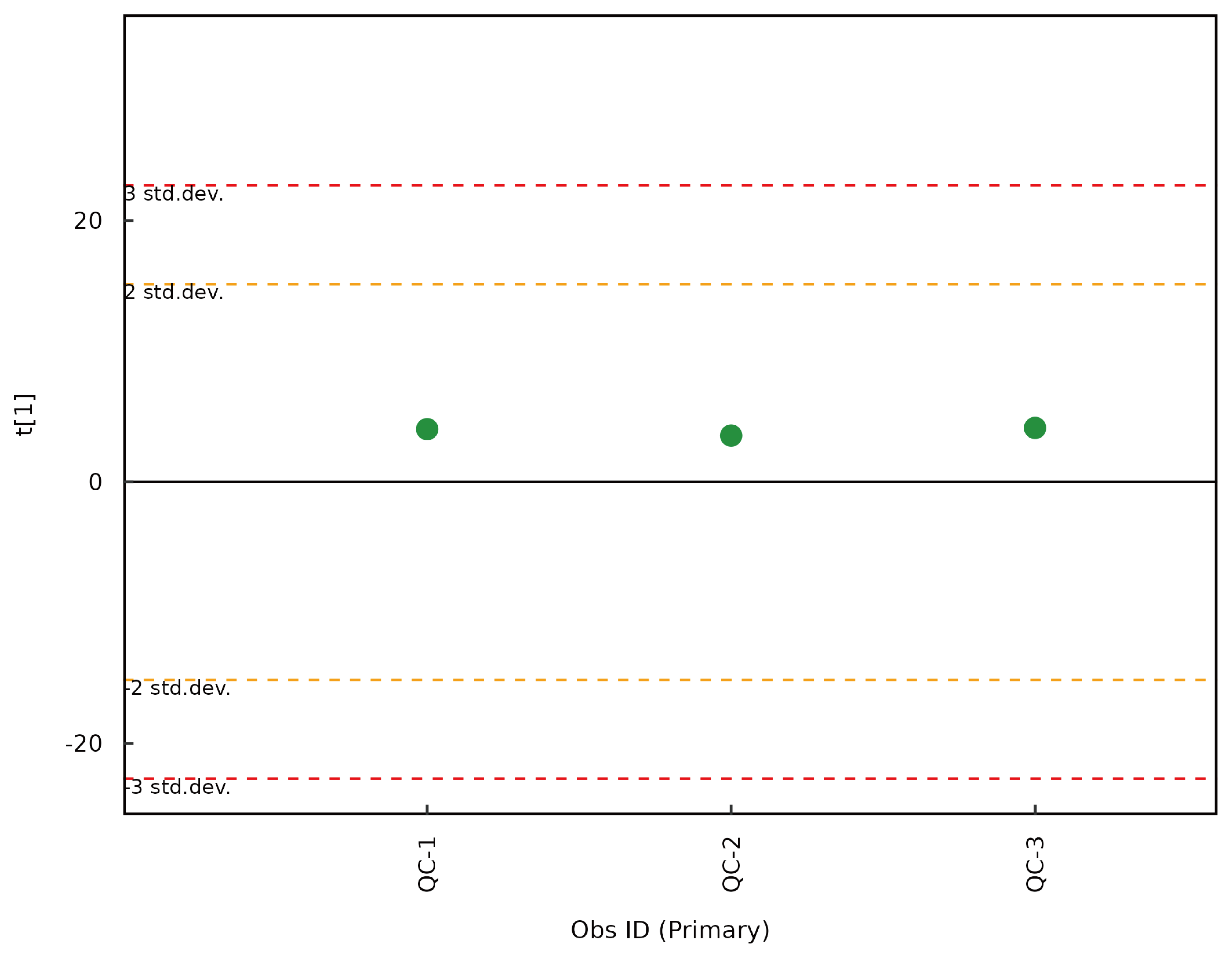


**Supplementary Figure 5.** Multivariate Control Chart of QC Samples


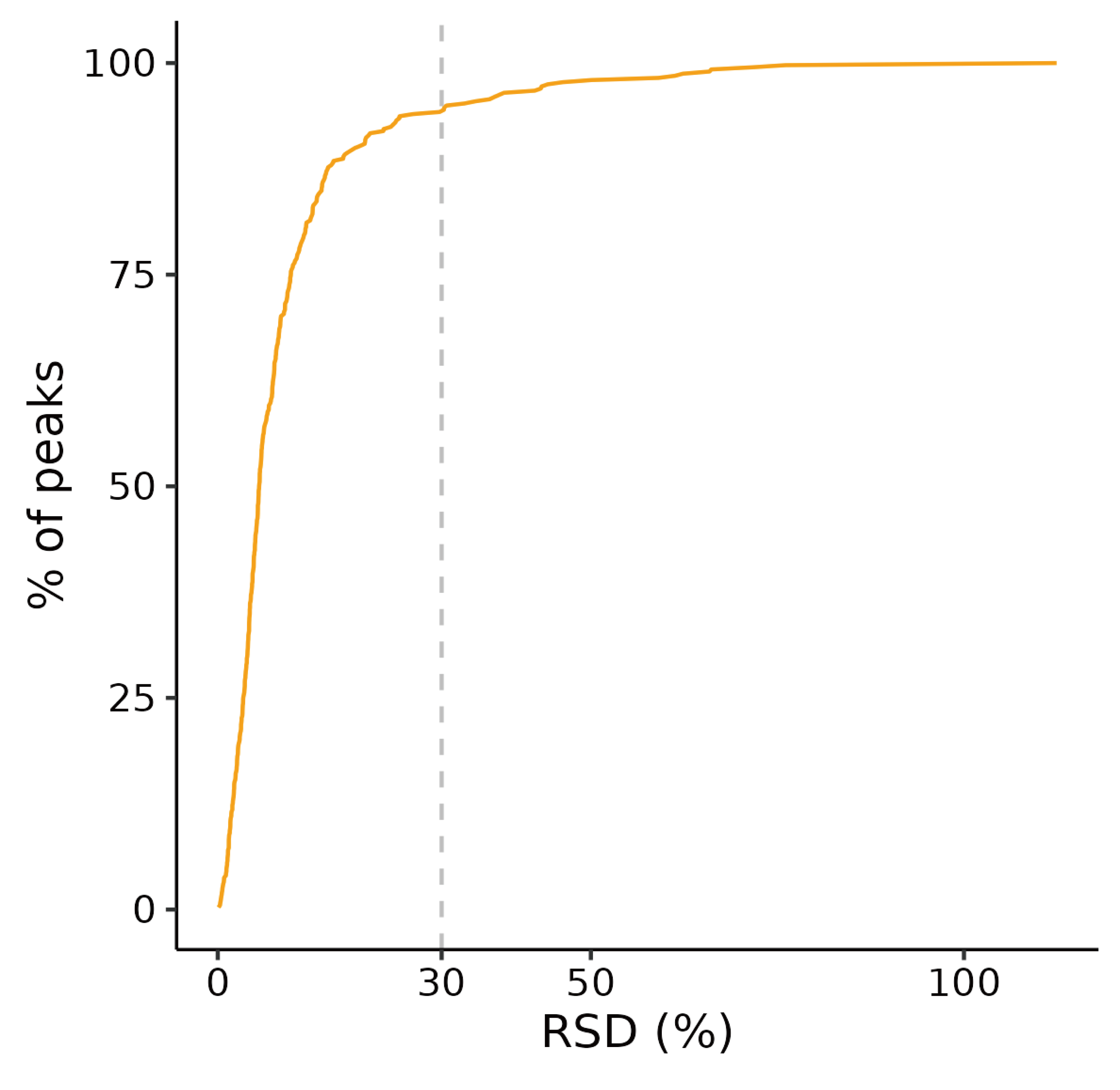


**Supplementary Figure 6.** Relative standard deviation of QC samples
